# A Highly Active Triterpene Derivative Capable of Biofilm Damage to Control *Cryptococcus* spp.

**DOI:** 10.3390/biom9120831

**Published:** 2019-12-05

**Authors:** Maria E. Krummenauer, William Lopes, Ane W. A. Garcia, Augusto Schrank, Simone C. B. Gnoatto, Daniel F. Kawano, Marilene H. Vainstein

**Affiliations:** 1 Centro de Biotecnologia, PPGBCM, Universidade Federal do Rio Grande do Sul, Porto Alegre, Rio Grande do Sul 91501-970, Brazil; mekrummenauer@gmail.com (M.E.K.); lopeswlm@gmail.com (W.L.); awichine@gmail.com (A.W.A.G.); argusto@gmail.com (A.S.); 2 Faculdade de Farmácia, Universidade Federal do Rio Grande do Sul, Porto Alegre, Rio Grande do Sul 90610-000, Brazil; simone.gnoatto@ufrgs.br; 3 Faculdade de Ciências Farmacêuticas, Universidade Estadual de Campinas, Campinas, São Paulo 13083-871, Brazil; dkawano@unicamp.br

**Keywords:** triterpenes, *Cryptococcus* spp., biofilm modulation, antifungals

## Abstract

*Cryptococcus neoformans* is an encapsulated yeast responsible for more than 180,000 deaths per year. The standard therapeutic approach against cryptococcosis is a combination of amphotericin B with flucytosine. In countries where cryptococcosis is most prevalent, 5-fluorocytosine is rarely available, and amphotericin B requires intravenous administration. *C. neoformans* biofilm formation is related to increased drug resistance, which is an important outcome for hospitalized patients. Here, we describe new molecules with anti-cryptococcal activity. A collection of 66 semisynthetic derivatives of ursolic acid and betulinic acid was tested against mature biofilms of *C. neoformans* at 25 µM. Out of these, eight derivatives including terpenes, benzazoles, flavonoids, and quinolines were able to cause damage and eradicate mature biofilms. Four terpene compounds demonstrated significative growth inhibition of *C. neoformans.* Our study identified a pentacyclic triterpenoid derived from betulinic acid (LAFIS13) as a potential drug for anti-cryptococcal treatment. This compound appears to be highly active with low toxicity at minimal inhibitory concentration and capable of biofilm eradication.

## 1. Introduction

The increased frequency and drug resistance of invasive fungal infections is an expanding public health problem worldwide, which has been neglected over the years [1]. It is estimated that 1.2 billion people worldwide suffer from such diseases [2]. Research on cryptococcosis, in particular, receives less than 0.5% of the total global research funding, and is classified by the G-Finder survey amongst the most poorly funded diseases [3].

*Cryptococcus neoformans* is an encapsulated yeast responsible for more than 180,000 deaths per year [4]. After the inhalation of spores, the yeast reaches the human lungs and efficiently disseminates to the brain, causing meningitis in immunosuppressed hosts [5]. *C. neoformans* also grows as a biofilm in architectural flower-like clusters [6]. The increased resistance of *C. neoformans* to antimicrobial therapies and host immune mechanisms is associated with its biofilm formation capacity [7].

The standard therapeutic approach against cryptococcosis is a combination of amphotericin B with flucytosine (also known as 5-fluorocytosine) [8]. The high mortality rates of cryptococcosis are associated with poor and late diagnosis, drug resistance, and low availability of treatments [9]. The estimated cost of 15 days of hospitalization for the intravenous treatment of cryptococcosis with liposomal amphotericin B can reach up to €20,000 in Europe [10], a prohibitive cost for the vast majority of patients and public health services. In countries where cryptococcosis is most prevalent, 5-fluorocytosine is rarely available [8], the intravenously treatment demands medical infrastructure, and amphotericin B is nephrotoxic [11]. Fluconazole is frequently used as an alternative treatment, although emerging resistance to this antifungal was described in different strains of *Cryptococcus* spp. and *Candida* spp. [12,13]. In the last 30 years, the last class of antifungals approved for clinical use was echinocandins in 2002, which also proved to be inefficient for *C. neoformans* [14].

Natural products may have potential for the development of new classes of drugs with high activity, low toxicity, and the capacity to undergo chemical optimizations [15]. Ursolic acid (UA) and betulinic acid (BA) are pentacyclic triterpenes extracted from natural sources, described as a promising antimicrobial, anticancer, and anti-inflammatory molecule [16,17]. The activity of semisynthetic derivatives of pentacyclic triterpenes against *Staphylococcus aureus* biofilm formation and bacterial growth has been reported [18].

Considering the need for new antifungals, herein, we aimed to find new molecules with anti-cryptococcal activity in a collection of 66 semisynthetic derivatives. Our study identified a pentacyclic triterpenoid derived from BA (LAFIS13) as an efficient future therapeutic molecule, with high activity, low toxicity, and capability to modulate biofilm development.

## 2. Materials and Methods

### 2.1. Strains and Growth Conditions

*C. neoformans* strain H99 (serotype A), B3501 (serotype D) and *Cryptococcus gattii* strain R265 (serotype B) were maintained in YPD agar. For capsule size determination, *C. neoformans* H99 was cultivated in minimal medium composed of MgSO_4_ (10 mM), KH_2_PO_4_ (29 mM), glycine (13 mM), glucose (15 mM), and thiamine-HCl (3 µM), pH 5.5, for 72h at 37 °C in 5% CO_2_. The cell line J774.16 (murine macrophages) was maintained in Dulbecco’s Modified Eagle Medium (DMEM) supplemented with 10% fetal bovine serum (FBS) at 37 °C in 5% CO_2_.

### 2.2. Screening of a Semisynthetic Compounds Collection for Biofilm-Damaging Molecules

*C. neoformans* B3501 cells can adhere to a surface and form biofilms. Biofilm formation is related to increased drug resistance in cryptococcosis treatment [19]. We evaluated a collection of 66 semisynthetic compounds from different sources to select molecules and chemical classes able to cause damage to mature biofilms of *C. neoformans* B3501. Each compound was diluted to 0.1 M in DMSO (dimethyl sulfoxide) and stored at −20 °C until further use. The compounds were diluted in MilliQ water to a final DMSO concentration <0.1%. To test biofilm damage caused by each compound, the samples were tested at a concentration of 25 µM. *C. neoformans* B3501 was grown overnight in YPD broth, washed twice with PBS, and 10^7^ cells were suspended in 100 µL of RPMI 1640 and added to each well. The yeast was incubated for 48 h at 37 °C. The mature biofilm was washed twice with PBS to remove the planktonic cells and then incubated with the compounds in RPMI 1640. For relative growth analysis, the optical density at 600 nm (OD_600_) was read. To analyze the relative biofilm damage, the crystal violet assay was performed, and the optical density at 570 nm was read using a SpectraMax i3x microplate reader.

### 2.3. Analysis of Antifungal Activity

The values of minimum inhibitory concentrations (MICs) for all species were determined using the protocol established by the European Committee on Antimicrobial Susceptibility Testing (EUCAST) with minor modifications [20]. The selected compounds were serially diluted (437.9 to 1 µM) in RPMI 1640 (pH 7.2; 2% glucose) buffered with MOPS (3-(N-morpholino) propanesulfonic acid). The microplates were incubated at 37 °C for 48 h. The MIC values corresponded to the lowest concentration able to inhibit fungal growth.

### 2.4. Evaluation of the Main Virulence Determinants of C. neoformans Treated with the BA Active Derivative N-{3-[4-(3-Aminopropyl)piperazinyl]propyl}-3-O-acetylbetulinamide (LAFIS13)

Pigmentation, urease activity, and capsule formation of *C. neoformans* were evaluated when the yeast was treated with the BA active derivative at MIC x 0.5, MIC x 0.25 [21], or subinhibitory concentration. Melanin production was visually analyzed by plating 10^5^ cells of the H99 strain in minimal medium agar with L-dopa (1 mM) and incubating with the BA active derivative for 72 h at 37 °C. Urease activity was evaluated according to standard protocols with minor modifications [22]. H99 cells (10^8^) and the BA active derivative were incubated in rapid Robert’s urea broth (RUH) for 4 h at 37 °C in a rotatory shaker at 700 rpm. The *C. neoformans* cells were centrifuged for 3 min at 5000 rpm. The supernatant was collected and diluted with MilliQ water. The absorbance at 560 nm (OD_560_) was read. All readings below 0.3 indicated ureolysis. For capsule measurements, *C. neoformans* cells were incubated in minimal medium with the BA active derivative for 72 h at 37 °C in 5% CO_2_. The cells were stained with India ink and analyzed by light microscopy; the cells and the capsule size of 50 cells per condition were measured using the ImageJ software (NIH) [23].

### 2.5. Analysis of Synergistic Effects

The synergistic activity of the BA active derivative and the standard drugs amphotericin B and fluconazole, against *C. neoformans* H99 was determined on the basis of the calculation of the fractional inhibitory index (FIC). The BA active derivative (denominated drug A) was serially diluted (17.1 to 2.3 µM) in 96-well plates. The standard antifungals (denominated drug B) were serially diluted (8 dilutions) from 16 to 0.125 µg/mL (amphotericin B) or from 64 to 0.5 µg/mL (fluconazole) [20]. FIC was defined as:

$$\frac{MICcombined}{MICdrugAalone}\;+\;\frac{MICcombined}{MICdrugBalone}$$

Synergism was categorized as follows: synergistic effect, FIC < 0.5; no effect, FIC 0.5–4; antagonist effect, FIC > 4 [24].

### 2.6. Scanning Electron Microscopy

To analyze the ultrastructure of *C*. *neoformans* B3501 after the treatment with the BA active derivative (291 µM), biofilm damage and capsule formation assays were performed. Scanning electron microscopy preparation was performed according to Lopes and Vainstein, 2017 with minor modifications [6]. The wells were washed three times with PBS, and cryptococcal adherent cells were fixed with 500 µL of 2.5% glutaraldehyde in 0.1 M cacodylate for 1 h at room temperature. Then, the wells were washed twice in post-fixative solution (0.1 M cacodylate, 2 mM MgCl_2_, and 0.2 M sucrose). The samples were serially dehydrated in alcohol (30%, 50%, and 70%, for 5 min, then 95% and twice with 100%, for 10 min), then subjected to critical point drying (EM CPD 200, Leica) and sputter-coating with gold–palladium (15–20 nm), and finally examined with the Auriga Zeiss or Zeiss MA10 microscope. Microscopic fields were randomly selected.

### 2.7. Cell Viability Assay

J774.16 peritoneal macrophages were seeded in 96-well culture plates at 37 °C and 5% CO_2_, containing DMEM supplemented with 10% (*v/v*) FBS, 100 U/mL penicillin, and 100 µg/mL streptomycin at a density of 10^5^ cells per well. After 18 h, the cells were exposed to different concentrations of the BA active derivative (11.4 µM and 291.9 µM) for 2, 4, and 24 h in DMEM supplemented with 10% FBS. The MTT (3-(4,5-dimethylthiazol-2-yl)-2,5-diphenyltetrazolium bromide) assay was performed as a colorimetric method for evaluating cell viability. After each incubation, 100 µL of MTT solution dissolved in PBS was added into each well, and the cells were incubated at 5% CO_2_ for 3 h at 37 °C. Subsequently, the supernatant was discarded, and 100 μL of DMSO was added to each well. Mitochondrial dehydrogenase enzymes in the viable cells reduce tetrazolium salts forming purple formazan, which can be quantified by reading the absorbance using a microplate reader (SpectraMax i3 - Molecular Devices). The results are expressed as the difference between the readings (570–630 nm) [25,26].

### 2.8. Toxicity in Galleria mellonella

To analyze the toxicity of LAFIS13 or amphotericin B, a single dose (10 µL) was injected into the last right proleg of *G. mellonella* larvae using a Hamilton syringe. Ten similar-sized larvae (about 300 mg) were selected. The final concentrations of the compounds in each larva ranged from 2.58 to 13 µg/g larvae (equivalent to 11.4 µM and 57.7 µM) for LAFIS13 and was 2 µg/g larvae for amphotericin B. Controls were injected with PBS only. Larval survival was monitored for 10 days. Insignificant differences in larval survival between the treated group and the control group indicated a lack of toxicity [27].

### 2.9. In Silico Predictions of the Pharmacokinects Profile of the BA Active Derivative

The structure of amphotericin B was downloaded from PubChem (https://pubchem.ncbi.nlm.nih.gov/), and the tridimensional structure of the BA active derivative (N-{3-[4-(3-aminopropyl)piperazinyl]propyl}-3-*O*-acetylbetulinamide) was built using the on-line version of Corina, which ascribes to 3-D structures pre-defined bond lengths and angles depending on the type of bond, type of atom, and hybridization state [28,29]. Corina also defines the most probable torsional angles according to the nature of the structure (acyclic, small/medium rings, macro/polycyclic, etc.), being able to correctly reproduce a varied number of X-ray structures [29]. Molecular geometries were then refined using the AM1 semi-empirical force field with the Broyden–Fletcher–Goldfarb–Shanno (BFGS) algorithm [30]. The structure was considered optimized when its conformational energy gradient was below 0.001 kcal mol^−1^. Both the piperazine nitrogens and the primary amine of N-{3-[4-(3-aminopropyl) piperazinyl]propyl}-3-*O*-acetylbetulinamide, as well as the carboxylic acid/amino group of amphotericin B were assumed as ionized at the physiological pH for the subsequent calculations. Such protonation states at the physiological pH were predicted using the MarvinSketch 16.5.2.0, 2016, ChemAxon (http://www.chemaxon.com).

Molecular Discovery VolSurf Plus 1.0.4 was then used to estimate the main pharmacokinetic parameters of the BA active derivative: distribution coefficient, hydrosolubility, plasma proteins binding, CYP3A4 metabolic stability, Caco-2 permeability, and blood–brain barrier (BBB) permeability. The software calculates molecular descriptors based on the three-dimensional GRID molecular interaction fields and has been successfully used to predict the ADMETox properties of a number of drugs [31].

The hydrosolubility was expressed as −Log[Soly], where Soly is the solubility in water (mol/L) at 25 °C. Compounds are predicted to have a poor solubility when −Log[Soly] is lower than −4, a medium solubility when −Log[Soly] is between −4 and −1, and a high solubility when −Log[Soly] is equal or higher than −1. The cytochrome P450 3A4 (CYP3A4) metabolic stability estimates the final concentration of a compound incubated for 60 min with a fixed concentration of the enzyme at 37 °C, whereby compounds with a final concentration greater than or equal to 50% are considered stables. Caco-2 cell permeability was expressed using transformed values, according to which a positive index indicates that the compound is well absorbed, and a negative indicates that the compound is poorly absorbed. Compounds are assumed as brain-penetrating for LogBB > 0.5, with moderate permeation for 0.5 ≤ LogBB < 0, with reduced ability to cross the BBB for 0 ≤ LogBB < −0.3, or very little brain-penetrating when LogBB < −0.3 [31].

The software Meteor 2.0 was used to predict the metabolic pathways of the BA active derivative. Simulations included phase I and II biotransformations processed until the 3^rd^ generation of metabolites, where all the molecules are conjugated or display low log P values, being more likely to be excreted. The analysis of toxicophoric groups regarding the parent structure and their proposed metabolites was carried out using the software Derek 2.0 in order to model the toxicity endpoints, including carcinogenicity, mutagenicity, genotoxicity, skin sensibilization, teratogenicity, airways hyperreactivity, hepatotoxicity, neurotoxicity, among others. Meteor and Derek identify metabolic reactions and toxicophoric groups (structural moieties in a specific compound that can exert toxic effects), respectively, through a knowledge-based system wherein structure–property correlations are searched from a database built from relevant literature concerning metabolic and toxicological data [32].

### 2.10. Statistical Analyses

Statistics were obtained with GraphPad Prism 6.0 software. To analyze toxicity in the *G. mellonella* survival curve, the Logrank test for trend and the Gehan-Breslow-Wilcoxon test were used. The variance two-way ANOVA was carried out using Tukey’s comparisons for minimal inhibitory concentrations. One-way ANOVA performed using Dunnett’s multiple comparisons was used for the minimal biofilm eradication concentration assay.

## 3. Results

### 3.1. Selection of the BA Active Derivative LAFIS13 as a Potential Agent against Cryptococcal Biofilm

**Figure 1:**
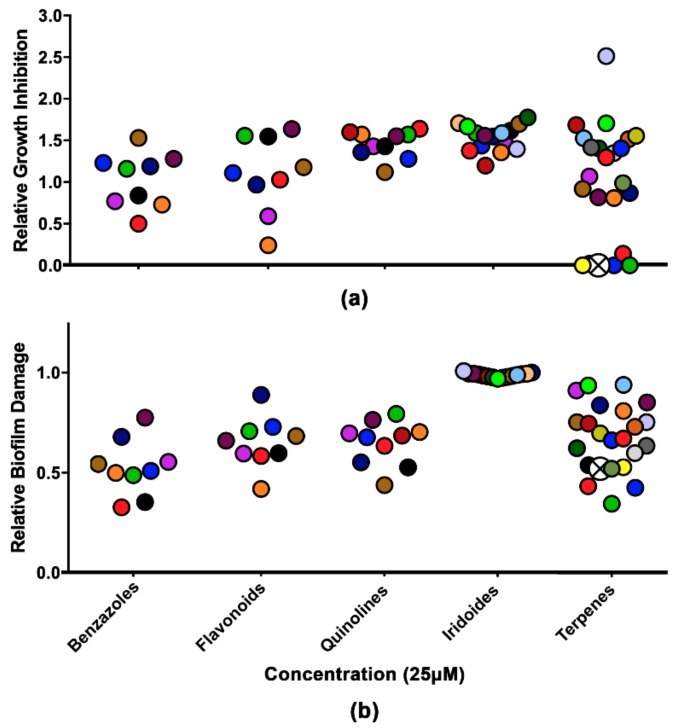
Analysis of growth inhibition and biofilm damage in the presence of molecules from the collection of semisynthetic compounds. (**a**) Relative growth inhibition. (**b**) Relative biofilm damage measured by crystal violet staining. The results were normalized using *Cryptococcus neoformans* without the compounds. The colors represent the same compound in the two experiments. Crossed white circles indicate LAFIS13. All molecules were used at a concentration of 25 µM.

The collection of 66 compounds was tested against mature biofilms of *C. neoformans* B3501 at 25 µM, and the relative yeast growth was quantified. The terpenes demonstrated complete inhibition of *C. neoformans* among the tested chemical classes. Biofilm eradication was seen for terpenes, benzazoles, flavonoids, and quinolines, being able to cause biofilm damage, with a threshold of 50% (Figure 1).

Derivatives of quinolines [33], flavonoids [34], and terpenes have been described with antimicrobial activities. It has already been demonstrated in *Candida albicans* that terpenes have antifungal and antibiofilm effects [35]. LAFIS13, a pentacyclic triterpene, was selected among the other terpenes due to its recently described promising properties and the absence of a negative impact on hematological and biochemical parameters [36]. LAFIS13 was obtained as described previously [37]. The chemical structure was confirmed using full spectroscopy data (IR, ^1^H, ^13^C NMR, HR-EI-MS, and elemental analysis data), and was consistent with that previously described.

### 3.2. Minimal Biofilm Damage Concentration of LAFIS13

**Figure 2:**
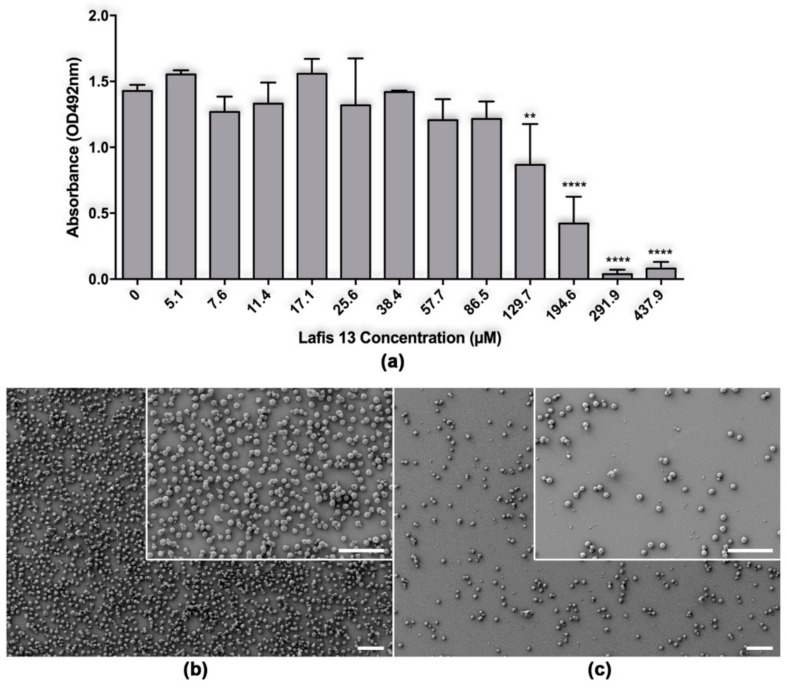
Effect of LAFIS13 on mature biofilms of *Cryptococcus neoformans*. (**a**) Metabolic activity of untreated and LAFIS13-treated *C. neoformans* strain B3501 biofilms measured by the XTT reduction assay. Mature biofilms were incubated with various concentrations (5.1 to 437.9 µM) of LAFIS13 for 24 h; each biofilm damage was compared to that of a biofilm incubated without the triterpene. Bars are the averages of three XTT measurements, and brackets denote standard deviations. **, *p* < 0.01 and ****, *p* < 0.0001 in comparing the untreated and LAFIS13-treated groups. (**b**) Scanning electron microscopy images of untreated *C. neoformans* B3501 biofilm formed on glass coverslips. (**c**) SEM image of *C. neoformans* B3501 biofilm treated with 291 µM shows biofilm eradication. Scale bar: 40 µm.

*C. neoformans* biofilms are described as resistant to azoles and other antifungal drugs at high concentrations [19]. The resistance of biofilms to conventional treatment is important due to the increasing use of ventriculoperitoneal shunts to manage intracranial hypertension [7]. LAFIS13 at the concentration of 129.7 or higher caused significant damage to mature biofilms of *C. neoformans* B3501 (Figure 2a).

SEM examination was used to visualize differences in the microarchitecture between control and LAFIS13-treated *C. neoformans* biofilms and indicated that LAFIS13 at 291 µM eradicated established biofilms (Figure 2a,b).

### 3.3. LAFIS13 is Fungicidal against Cryptococcus spp.

To test the fungicidal activity of LAFIS13, the minimal inhibitory concentration test was performed. Observed MIC and minimum fungicidal concentration (MFC) values were different for *C. neoformans* and *C. gattii*. The MIC and MFC values for *C.* *neoformans* B3501 and H99 were both 11.4 µM, while those for *C. gattii* R265 were 7.6 µM.

### 3.4. Evaluation of Potential Synergism between LAFIS13 and Standard Antifungals

Although the need for new antifungal therapies is clear, drug research and development are costly. Synergy between drugs could be an alternative approach. To evaluate whether the association of LAFIS13 with amphotericin B or fluconazole results in improved activity against *C. neoformans* H99, the FIC index was calculated (Table 1). We found that LAFIS13 demonstrated a synergistic effect against *C. neoformans* when combined with fluconazole, but no synergy was found for amphotericin B.

### 3.5. Effects on Capsule Size, Urease Production, and Melanization of Cryptococcus Neoformans

To evaluate whether LAFIS13 could modulate virulence determinants in *Cryptococcus* spp., capsule induction, urease and melanin production were evaluated in the presence of the triterpene. LAFIS13 was unable to modulate the virulence determinants tested.

### 3.6. LAFIS13 is Non-Toxic at MIC

**Figure 3:**
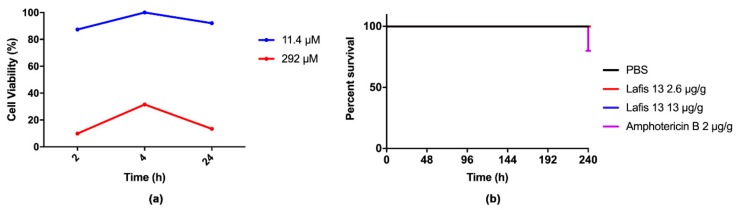
In vitro and in vivo toxicity assays. (**a**) Cytotoxicity was analyzed using J774.16 peritoneal macrophages; the compound was not toxic at 11.4 µM; (**b**) survival curve of *Galleria mellonella* during 10 days. No deaths were observed in the LAFIS13 group.

To evaluate the toxicity of LAFIS13, the MTT cell viability assay was performed in mammalian cells. Our results showed that the viability of J774.16 peritoneal macrophages was maintained after treatment with LAFIS13 at MIC of 11.4 µM. However, cytotoxicity was observed upon acute exposure and chronic exposure to LAFIS13 at 291.9 µM concentration (Figure 3a). The toxicity of LAFIS13 in *G. mellonella* was evaluated for 10 days. The survival curve did not demonstrate any significant differences when compared to the control survival curve (PBS), which indicates that the compound is not toxic (Figure 3b).

### 3.7. In Silico Predictions of LAFIS13 Pharmacokinects Profile

**Figure 4:**
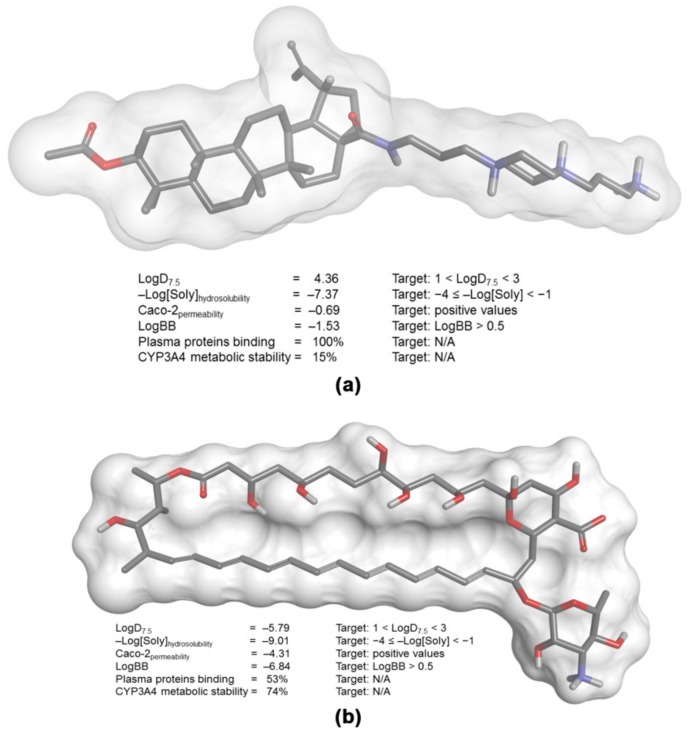
Main pharmacokinetic parameters calculated on the basis of the 3-D structure of (**a**) LAFIS13 and (**b**) amphotericin B.

**Figure 5:**
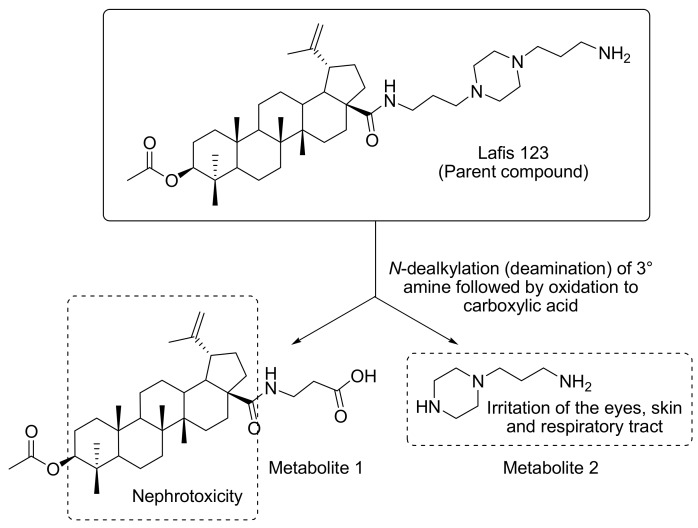
Main toxicophore endpoints and related metabolic pathway predicted for LAFIS13.

More than 65 years after its discovery, amphotericin B (with or without 5-flucytosine) remains the gold standard for the therapy of cryptococcosis, a very alarming fact considering that more than 30% of the patients experience drug-related nephrotoxicity [38,39]. Therefore, the challenge is the development of better-tolerated and metabolically stable drugs that are still effective in the treatment of a very life-threatening condition. In this context, to assess the pharmacokinetics profile of LAFIS13, we performed in silico predictions based on the 3-D structure of the semisynthetic compound (Figure 4a), which were compared with the corresponding parameters of the reference drug amphotericin B (Figure 4b), [31]. Concerning the most probable metabolites of LAFIS13, besides the predicted hydrolysis of the acetyl group and the subsequent oxidation of the resulting free secondary hydroxyl to a ketone group, LAFIS13 is expected to undergo oxidative *N*-dealkylation to yield piperazine-1-propylamine [3-(piperazin-1-yl)propan-1-amine] as a metabolite (Figure 5, metabolite 2).

## 4. Discussion

Historically, natural products have been a source for drug design. Natural products and their derivatives continue to undergo clinical trials [40,41]. An analysis of FDA-approved drugs between 1981 and 2010 revealed that 34% of these drugs are based on small molecules derived from natural products [42,43]. Since 2000, 22 new antibiotics have been approved for the treatment of human infectious diseases, 3 of which represent new classes originating from natural products [44]. Considering the chemical importance of natural sources, semi-synthesis allows the improvement of activity for derivatives generated from natural products. *C. neoformans* is a ubiquitous yeast that mostly affects regions with limited health infrastructure resources. The slow mobility of the standard treatment for cryptococcosis is associated with high expenses and limited access [8]. Since the socioeconomic scenario behind cryptococcosis is characterized by high mortality, alternatives to the treatment are needed. Terpenes are widely described as compounds with antimicrobial, anti-inflammatory, and anticancer activities [16,17]. This study selected a pentacyclic triterpene, LAFIS13, obtained from BA, to investigate its toxicity and antifungal and antibiofilm properties.

*C. neoformans* infection is based on virulence determinants, which cause difficulties in the management of cryptococcosis. The fact that LAFIS13 was not able to modulate the main virulence factors suggests that its mechanism of action does not concern the pathogenic pathways previously described. Studies have demonstrated that the drugs capable of modulating *C. neoformans* biofilms were effective only at high concentrations [45]. In our study, LAFIS13 was an efficient agent against *C. neoformans* mature biofilms. The minimum LAFIS13 concentration required for antifungal activity against planktonic cells (11.4 µM) was much lower than the doses required for activity against mature biofilms (291 µM).

Synergy between drugs and molecules could be an alternative approach to drug research and development. The combination of amphotericin B and flucytosine is an example of improved efficacy [46]. LAFIS13 demonstrated synergism when combined with fluconazole.

The viability of J774.16 was maintained after treatment with LAFIS13 at the inhibitory concentration of 11.4 µM; however, cytotoxicity was observed at the high concentration of 291.9 µM. LAFIS13 was tested in *G. mellonella* for 10 days, and the survival curve indicated less toxicity compared to amphotericin B. The toxicity profile for LAFIS13 may still be considered more desirable when compared with that of the standard treatment, i.e., amphotericin B, which normally causes renal toxicity (up to 80%), electrolyte wasting (K^+^ and Mg^++^), normochromic/normocytic anemia, central nervous system (CNS) toxicity, as well as, occasionally, life-threatening reactions such as anaphylaxis, acute hepatic failure, seizures, ventricular fibrillation, and cardiac arrest [47].

Concerning the in silico simulations, the distribution coefficient of LAFIS13 (LogD_7.5_ = 4.36) indicates a relative lipophilic behavior, with consequently poor water solubility (−Log[Soly] = −7.37). Although amphotericin B displays a distribution coefficient that suggests a more hydrophilic behavior (LogD_7.5_ = −5.79), its overall water solubility is also poor (−Log[Soly] = −9.01); in fact, this antifungal is defined as a poorly soluble and permeable drug, and its solubilization is only achieved in deoxycholate-based micelles (Fungizone^®^) designed for intravenous administration [48].

Because distribution coefficient of both compounds is somewhat distant from the ideal range predicted for drugs with good bioavailability (1 < LogD_7.5_ < 3) [49], it is also expected that they will display poor permeability in human colon adenocarcinoma cells (as expressed by the negative indices for Caco-2 permeability), which represent an in vitro model for the estimation of in vivo absorption from intestinal epithelial cells [31,49]. For the same reason, both LAFIS13 and amphotericin B would lack the ability to cross the blood-brain barrier and, in fact, the antifungal has been shown to poorly cross the blood-brain barrier [47]. However, as several organism-associated molecules (e.g., interleukin-1 beta, tumor necrosis factor alpha, lipopolysaccharide (LPS), etc.) produced during meningitis may increase the permeability of drugs across the blood–brain barrier due to the disruption of the endothelial layer integrity (Pyrgos et al., 2010), both LAFIS13 and amphotericin B may be considered viable alternatives to treat cryptococcosis. Because of its relative high lipophilicity, LAFIS13 would display a high affinity for plasma proteins (protein binding = 100%) and is expected to undergo extensive first-pass metabolism catalyzed by the CYP450 system (CYP3A4 metabolic stability = 15%), possibly producing toxic metabolites. Although amphotericin B is not predicted to display high affinity for plasma proteins in its free form, amphotericin B deoxycholate is highly protein-bound (>95%) [50], and the drug does not seem to be extensively metabolized in humans [51], so drug toxicity is expected to be mostly associated with the parent compound.

Regarding the probable metabolites of LAFIS13, the drug is expected to yield piperazine-1-propylamine [3-(piperazin-1-yl)propan-1-amine] as a metabolite (Figure 5, metabolite 2). Alkyl diamines are known to cause irritation of the eyes, skin, and upper respiratory tract [52], and the same effects are also observed for piperazines [53]. Accordingly, there is a risk for the development of skin rashes in humans after oral administration of LAFIS13, as previously reported for piperazine and ethylenediamine [54].

We must highlight, however, that this may be considered a minor toxicity issue, as we previously demonstrated that the drug is safe for experimental animals when administrated at moderate doses. In this regard, LAFIS13 showed high toxicity at doses of 2000 and 300 mg/kg and caused the death of all the exposed animals. However, at 50 mg/kg, LAFIS13 was not lethal, and no signs of toxicity were observed [36]. According to the OECD-423 protocol, LAFIS13 LD_50_ ranges between 300 and 50 mg/kg; thus, it fits the class 3 of chemical agents, as reported by the Globally Harmonized Classification System (GHS).

An additional potential risk associated with the use of LAFIS13 is the development of nephrotoxicity. Since alicyclic hydrocarbons with LogP > 3.5 are known to accumulate in the renal cortex causing nephropathy and cortical tumors [55], the lipophilic core in both the parent drug and Metabolite 1 (Figure 5) may represent a risk. However, it can be simply suppressed by the conversion of the free-base form of the drug into a salt, which, after proper selection of the counterion, will increase the drug’s hydrosolubility and, accordingly, reduce the risk of nephropathies [56].

The same does not apply to amphotericin B, for which nephrotoxicity develops from direct damage of the renal tubules and from constriction of the renal vasculature [39].

Although LAFIS13 is not completely devoid of toxicity, the therapeutic potential of this drug must be assessed comparatively to the other clinically available treatments, particularly amphotericin B, which remains as the gold standard for the therapy of cryptococcosis [38]. Drugs as fluconazole and some newer azoles, for example, are very safe and can be orally administered but are also typically inferior in effectiveness to the standard treatment even at very high doses [57]. Therefore, the ultimate decision about whether a particular compound should be further developed as a therapeutic alternative for the management of cryptococcosis must be based on a balance between its intrinsic fungicidal activity and its potential toxicity. In this respect, we are convinced that LAFIS13 represents a viable alternative. Naturally, as in silico predictions should not be assumed as a direct replacement for in vivo results, unless one observes a great confidence in the prediction [58], additional in vitro and in vivo studies must be performed to confirm (or disprove) the toxicity predicted for the drug.

## 5. Conclusions

LAFIS13 inhibits *C. neoformans* growth and causes damage to its biofilm. Toxicity assays were performed, and LAFIS13 did not demonstrate toxicity at the MIC. However, cytotoxicity was observed at 291.9 µM. The toxicity profile of LAFIS13 is favorable when compared with that of amphotericin B. The selected molecule demonstrated potential as an anti-*Cryptococcal* treatment, but more aspects should be explored through in vivo experiments in order to confirm the pharmacokinetics profile of LAFIS13.

## Figures and Tables

**Table 1:** Impact of the association of LAFIS13 (drug A) with amphotericin B (AmB) or fluconazole (Flu) (drug B) on the antifungal activity against *C. neoformans* H99.

	(Drug A)			(Drug B)		
	MIC alone (µg/mL)	MIC combined (µg/mL)		MIC alone (µg/mL)	MIC combined (µg/mL)	FIC ^1^
**LAFIS13**	7.76	0.648	**AmB**	**0.41**	0.33	0.89
**LAFIS13**	7.76	0.648	**Flu**	**7.00**	1.58	0.31

^1^ Fractional inhibitory concentration index (FIC); synergistic: FIC ≤ 0.5; indifferent FIC > 0.5–4; antagonist: FIC > 4 [24].
